# Safe Utilization and Ecological Restoration of Heavy Metal Polluted Farmland: Latest Strategies for Remediation

**DOI:** 10.3390/toxics14020154

**Published:** 2026-02-03

**Authors:** Bin Guo, Ying Feng

**Affiliations:** 1 State Key Laboratory for Quality and Safety of Agro-Products, Institute of Environment, Resource, Soil and Fertilizers, Zhejiang Academy of Agricultural Sciences, Hangzhou 310021, China; 2 MOE Key Laboratory of Environment Remediation and Ecological Health, College of Environmental and Resource Sciences, Zhejiang University, Hangzhou 310058, China; yfeng@zju.edu.cn

## 1. Introduction

Heavy metal pollution of farmland has emerged as a pressing global environmental challenge, which threatens food security, ecological integrity, and human health [1]. Innovative strategies for the safe utilization of heavy metals and the ecological restoration of farmland are required to combat the persistence, mobility, and bioaccumulation of heavy metals such as cadmium (Cd), lead (Pb), and copper (Cu) in agricultural ecosystems [2]. This Special Issue consolidates eight original research articles that address key aspects of heavy metal polluted farmland management, covering contamination characterization, remediation technologies, microbial responses, and predictive modeling. The collected studies provide valuable insights into the complex interactions between heavy metals, soil, plants, and microorganisms, offering practical solutions for sustainable farmland management.

Over the past decades, research on heavy metal pollution control has developed from single-factor remediation to comprehensive ecological restoration. Traditional physical and chemical remediation methods are often costly and environmentally disruptive, highlighting the need for eco-friendly alternatives [3,4]. Phytoremediation, soil amendment, and microbial regulation have emerged as promising approaches, but their effectiveness varies with pollution types, soil properties, and regional conditions [5]. This Special Issue aims to consolidate cutting-edge research findings, identify knowledge gaps, and guide future directions in the field of heavy metal polluted farmland restoration.

## 2. Overview of Published Contributions

### 2.1. Contamination Characterization and Source Apportionment

Three studies focused on the temporal and spatial variation in heavy metal pollution and source identification, providing a scientific basis for targeted remediation.

Cao et al. (Contribution 1) investigated the temporal and spatial variation in eight toxic metals (As, Cd, Cr, Cu, Hg, Ni, Pb, Zn) in cultivated soil in Jiaxing, Zhejiang Province. The results demonstrated that compared with the early 1990s, Cd, Zn, Cu, Pb, Ni, and Cr concentrations increased by 86.2%, 27.2%, 41.1%, 45.6%, 4.94%, and 21.1%, respectively. Using local soil background values as the standard, all elements reached mild pollution levels, with Cd and Hg being the most severely contaminated. Industrialization, urbanization, and the use of metal-containing feed were identified as the main drivers of temporal variation, while soil types and crop cultivation methods dominated spatial differences.

Chen et al. (Contribution 2) employed principal component analysis (PCA) and positive matrix factorization (PMF) to identify sources of potentially toxic elements (PTEs) in agricultural soils of Yingtan City, Jiangxi Province. The average concentrations of Zn, Cu, Pb, Mo, and Cd exceeded local background values, with Cu and Cd showing the highest pollution levels. Four main sources were identified: mining activities, natural sources, copper smelting, and agricultural practices. The integration of PCA-PMF with field surveys effectively pinpointed pollution sources and specifically highlighted the role of mixed fertilizer application in Cd and Cu accumulation.

Wang et al. (Contribution 3) proposed a modified PCA-MLRD model integrated with grouped principal component analysis (GPCA) and GeoDetector for quantitative source apportionment in the Chang-Zhu-Tan region, Hunan Province. The study found that Cd and As were the main over-standard elements, with Cd exceeding the standard by 1.8 times on average and with an over-standard rate of 90%. Metal manufacturing factories contributed 30–44% to Cd pollution and 42–58% to As pollution, while soil parent material was the primary source of Pb (37–61%). Pollution pathways— such as atmospheric deposition, irrigation water, traffic emissions, and irrigation water—showed a more pronounced effect within 1000 m. The modified model improved accuracy by accounting for pollution processes, providing technical support for precise pollution control.

### 2.2. Phytoremediation and Plant Tolerance

Three articles explored the potential of phytoremediation using different plant species, emphasizing the selection of high-efficiency remediation plants and optimization of remediation effects.

Lu et al. (Contribution 4) conducted a two-year field trial on 23 chrysanthemum cultivars in Cd-contaminated soil in Zhejiang Province. Six cultivars (including marigolds) showed high Cd accumulation (average >0.6 mg per plant), with rhizosphere soil remediation taking only 4–5 years. Fourteen cultivars exhibited good multiple-cropping characteristics, and five (e.g., QX-yz) demonstrated high heavy metal tolerance. The application of bamboo vinegar significantly promoted Cd absorption in chrysanthemums, and an economic benefit analysis showed that planting dominant cultivars could generate income slightly higher than the local average.

Xu et al. (Contribution 5) compared the Pb tolerance and accumulation capacity of two industrial hemp varieties (Yunma1 (YM) and Shaanxi Industrial Hemp (SM)) under Pb stress. Pb mainly accumulated in the roots of both varieties (70–80%), with YM showing twice the Pb accumulation capacity of SM at high concentrations (5000 mg/kg). YM retained 70% of absorbed Pb in roots and exhibited strong tolerance (tolerance index > 90%) even at Pb concentrations exceeding 4000 mg/kg; whereas SM showed obvious toxicity symptoms. This research demonstrated the potential of industrial hemp to remediate Pb-contaminated mining soils.

Wang et al. (Contribution 6) evaluated the combined effects of Cd and F on lettuce growth and soil bacterial communities in co-contaminated yellow soil. Low concentrations of Cd and F had no significant effect on lettuce growth, but synergistic negative effects were observed when F exceeded 300 mg/kg and Cd exceeded 1.0 mg/kg. Lettuce accumulated Cd and F mainly in roots, with translocation factors (TrF) ranging from 0.313–0.941 to 0.499–0.754, respectively. This study highlighted the importance of considering combined pollution in remediation practices.

### 2.3. Soil Amendment and Immobilization Technologies

Two studies focused on soil amendment technologies, exploring the potential of waste-derived materials to immobilize heavy metal.

Cecire et al. (Contribution 7) investigated the use of rice husk as a sustainable amendment for heavy metal immobilization. Rice husk showed high retention capacities for Cu (100%), Cd (64%), and Mn (18%) under optimal conditions (particle size 90–300 µm, pH > 5.5, low buffer concentration). Pot experiments demonstrated that rice husk reduced heavy metal uptake by Lactuca sativa and Spinacia oleracea by 40–60% for Mn and Zn, and nearly 100% for Cr, Cu, Ni, Cd, and Pb. Factors such as abundance, low cost, and environmental friendliness make the material a promising option for soil remediation.

Rashid et al. (Contribution 8) developed machine learning models to predict Cd transformation and immobilization in biochar-amended soils. Three models (LSTM, BiGRU, and 5-layer CNN) were tested, with the 5-layer CNN achieving the highest accuracy (R^2^ = 0.956) in biochar-amended soils. Key factors that were identified as influencing Cd immobilization were as follows: soil pH, organic carbon (OC), cation exchange capacity (CEC), electrical conductivity (EC), and clay content. Biochar effectively reduced Cd availability by altering soil properties, providing a data-driven approach for the optimization of biochar application.

### 2.4. Microbial Responses and Ecological Restoration

One article focused on the response of soil microbial communities to heavy metal stress, shedding light on the ecological mechanisms of soil restoration.

Wang et al. (Contribution 6) studied the effect of Cd, F, and their combination on soil bacterial communities in lettuce rhizosphere. Proteobacteria was the dominant phylum (33.42–44.10%), and compartment (rhizosphere vs. bulk soil) was the primary factor driving community variation, followed by pollutant stress. F and Cd showed synergistic effects on bacterial communities, with the rhizosphere enriching specific taxa (e.g., Oxyphotobacteria, Subgroup 6) to enhance plant resistance. This study emphasized the role of soil microorganisms in mediating plant–pollutant interactions during remediation.

## 3. Key Insights and Future Directions

This Special Issue showcases the diversity and depth of research in heavy metal polluted farmland management and offers several key insights:

First, accurate source identification is the premise of effective remediation. The combination of multivariate statistical methods (PCA, PMF) and field surveys proves effective in distinguishing natural and anthropogenic sources, providing targeted guidance for pollution control. Second, phytoremediation demonstrates great potential when selecting appropriate plant species and optimizing cultivation strategies. Chrysanthemums and industrial hemp demonstrate both high remediation efficiency and economic benefits, addressing the long-standing problem of low profitability in phytoremediation. Third, soil amendments derived from agricultural waste (e.g., rice husk, biochar) offer sustainable and cost-effective options for heavy metal immobilization, aligning with the principles of circular economy. Fourth, the response of soil microbial communities to heavy metal stress plays a crucial role in ecological restoration; manipulation of rhizosphere microorganisms could further enhance remediation efficiency.

Despite these advances, several challenges and future directions remain:

1. Combined pollution remediation: Most studies focused on single or a few heavy metals, despite the fact that farmland is often contaminated by multiple PTEs. Future research should explore the mechanisms of combined pollution and develop synergistic remediation technologies [6].
2. Long-term field validation: Many studies are conducted under controlled conditions; long-term field trials are needed to verify the stability and durability of remediation effects under natural environmental fluctuations [7].
3. Integration of multiple technologies: The combination of phytoremediation, microbial remediation, and soil amendment could improve remediation efficiency. For example, the co-application of biochar and hyperaccumulators shows promising results [8].
4. Risk assessment and safe utilization: More research is needed to establish thresholds for the safe utilization of polluted farmland, integrating crop quality, soil health, and human health risks [9].
5. Digitalization and intelligent management: Machine learning models and digital tools should be further developed to optimize remediation strategies, predict pollution trends, and promote the traceability of agricultural products from polluted areas [10].

## 4. Conclusions

This Special Issue provides a comprehensive overview of the latest research progress in the safe utilization and ecological restoration of heavy metal polluted farmland. The collected studies cover contamination characterization, source identification, phytoremediation, soil amendment, microbial responses, and digital modeling, offering practical solutions and theoretical support for addressing this global challenge.

Notably, all studies are based on case studies from China, a country facing severe farmland heavy metal pollution due to rapid industrialization and intensive agriculture. The research findings not only contribute to solving local environmental problems but also provide valuable references for other regions with similar challenges. This Special Issue highlights the importance of an all-encompassing approach to the management of heavy metal polluted farmland, by integrating ecological principles, economic benefits, and technological innovations.

As we move forward, continuous research and innovation are essential to develop more efficient, sustainable, and cost-effective remediation technologies. It is also crucial to strengthen policy support, promote interdisciplinary collaboration, and enhance public awareness to achieve the dual goals of environmental protection and food security. We hope this Special Issue will inspire more researchers and practitioners to contribute to the safe utilization and ecological restoration of heavy metal polluted farmland, advancing towards a more sustainable and resilient agricultural system.

## Data Availability

Not applicable.
